# Low-Temperature Biosurfactants from Polar Microbes

**DOI:** 10.3390/microorganisms8081183

**Published:** 2020-08-03

**Authors:** Benjamin Trudgeon, Markus Dieser, Narayanaganesh Balasubramanian, Mitch Messmer, Christine M. Foreman

**Affiliations:** 1 Center for Biofilm Engineering, Montana State University, Bozeman, MT 59717, USA; benjamintrudgeon@gmail.com (B.T.); mitchmessmer@montana.edu (M.M.); cforeman@montana.edu (C.M.F.); 2 Department of Civil & Environmental Engineering, Montana State University, Bozeman, MT 59715, USA; 3 Department of Chemical & Biological Engineering, Montana State University, Bozeman, MT 59715, USA; 4 Department of Chemistry and Biochemistry, Montana State University, Bozeman, MT 59717, USA; b.narayanaganesh@gmail.com

**Keywords:** biosurfactant, di-rhamnolipid, bioremediation, Antarctic bacteria, cold temperature

## Abstract

Surfactants, both synthetic and natural, are used in a wide range of industrial applications, including the degradation of petroleum hydrocarbons. Organisms from extreme environments are well-adapted to the harsh conditions and represent an exciting avenue of discovery of naturally occurring biosurfactants, yet microorganisms from cold environments have been largely overlooked for their biotechnological potential as biosurfactant producers. In this study, four cold-adapted bacterial isolates from Antarctica are investigated for their ability to produce biosurfactants. Here we report on the physical properties and chemical structure of biosurfactants from the genera *Janthinobacterium*, *Psychrobacter*, and *Serratia*. These organisms were able to grow on diesel, motor oil, and crude oil at 4 °C. Putative identification showed the presence of sophorolipids and rhamnolipids. Emulsion index test (*E*_24_) activity ranged from 36.4–66.7%. Oil displacement tests were comparable to 0.1–1.0% sodium dodecyl sulfate (SDS) solutions. Data presented herein are the first report of organisms of the genus *Janthinobacterium* to produce biosurfactants and their metabolic capabilities to degrade diverse petroleum hydrocarbons. The organisms’ ability to produce biosurfactants and grow on different hydrocarbons as their sole carbon and energy source at low temperatures (4 °C) makes them suitable candidates for the exploration of hydrocarbon bioremediation in low-temperature environments.

## 1. Introduction

Surfactants, also known as surface-active agents, are commonly used in many different industries, including paints, textiles, detergents, health, and personal care products, as well as mineral and petroleum processing [1,2,3,4]. Surfactants, which may be natural or synthetic, are amphipathic compounds composed of a hydrophilic head, which is water-soluble, and a hydrophobic tail that is water insoluble. This chemical composition allows surfactants to reduce the interfacial tensions between liquids, solids, and gasses. Although highly effective in many industrial and remediation applications, synthetic surfactants tend to be toxic and can pose environmental hazards and biocidal activity [5]. For example, they have low biodegradability and can solubilize hydrophobic xenobiotics, affecting drinking water quality and wastewater treatment [6,7].

Biosurfactants are natural surfactants, produced primarily by microorganisms and plants [8,9,10]. As such, they are an attractive alternative to conventional surfactants, and are considered a key technology to develop in the 21st century [11]. Biosurfactants possess several advantages over their synthetic counterparts, including increased biodegradability, low toxicity, high-foaming properties, eco-friendliness, sustainable production, and high stability across extreme pH levels, temperatures, and salinity ranges [11,12,13,14,15,16]. Some of the most common types of biosurfactants are glycolipids, rhamnolipids, sophorolipids, trehalolipids, lipoproteins and lipopeptides, fatty acids, phospholipids, and polymeric structures, such as emulsan and liposan [11,13,17]. Akin to their synthetic counterparts, these different types of biosurfactants have broad commercial application, as they offer safer, more effective alternatives [8,17,18,19,20,21,22,23,24,25,26]. While the physiological functions and roles of biosurfactants in nature are not precisely defined, they are believed to promote the uptake and biodegradation of poorly soluble substrates, regulate biofilm structure and surface attachment/detachment, be involved in quorum sensing, and function as immune modulators and virulence factors [27]. Biosurfactants may also play a role as osmolytes [28], and glycolipid biosurfactants isolated from an Antarctic yeast have shown ice recrystallisation inhibitory activity [29].

In particular, bioremediation is an emergent strategy for cleaning up petroleum-contaminated environments [26,30,31]. Petroleum, a mixture of hydrocarbon-containing materials, remains a mainstay of current economic activity; however, this cornerstone role comes with some peril. Accidentally released hydrocarbons from leaks and spills, e.g., from onshore and offshore drilling, as well as the transportation of crude oil and refinery products, put many terrestrial and aquatic/marine environments at high risk. While leaks and spillage can be sporadic, they are nonetheless major environmental disasters. In the context of the Deepwater Horizon oil spill, $4 billion spent on mechanical clean-up removed ~25% of the oil spilled, with the majority of waste removal occurring naturally due to a deep-water bloom of bacteria [32], highlighting the monetary value associated with ecosystem services, such as bioremediation [33]. The ability to emulsify hydrocarbons by producing surface-active compounds is one of the most important characteristics of hydrocarbon-degrading bacteria. The most widely studied microbes for oil, diesel, and polycyclic aromatic hydrocarbon spill remediation are *Pseudomonas* spp., *Bacillus* spp., *Rhodococcus* spp., *Candida* spp., *Lactobacillus* spp., *Arthobacter* spp., and *Acinetobacter* spp. [31].

Many untapped oil reservoirs, current drilling efforts, and extensive distribution networks (including offshore drilling and subsea pipelines) are located in cold temperature environments. These regions are particularly vulnerable to spills, as low temperatures not only change the chemical properties and bioavailability of oil, but also slow down its biodegradation. Biosurfactants produced by model organisms, however, have typically been investigated at temperatures >20 °C [15,34,35,36,37], which are outside the temperature regime of the cold biosphere. Little attention has been paid to the quest for mining the biotechnological potential of biosurfactants produced by microorganisms from cold environments [28,38,39]. To help resolve this gap, the present study focused on biosurfactant production and potential hydrocarbon degradation of a suite of bacterial isolates from Antarctic environments, including genera previously unknown for biosurfactant production.

## 2. Materials and Methods

### 2.1. Sampling Locations of Microbial Isolates

**Figure 1:**
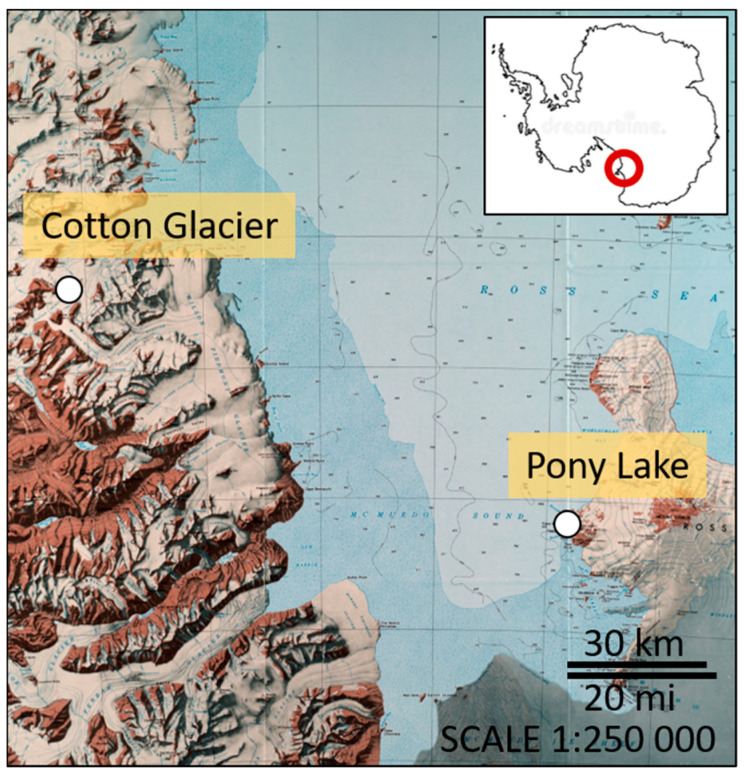
Map showing the location of the three sampling sites on Ross Island (Pony Lake) and the Transantarctic Mountain range (Cotton Glacier). (Credit: U.S. Geological Survey (USGS) Topographic Reconnaissance Series, 1986).

Pony Lake (PL) is a small (~120 m long, 70 m wide, and 1–2 m deep), brackish, and eutrophic lake located at Cape Royds (77°33′ S, 166°00′ E), Ross Island, Antarctica (Figure 1). The lake is ice covered or frozen solid to its base, except for a few weeks during mid-summer. Water temperatures ranging between 1.3–7.5 °C have been reported [40]. PL has no visible inflow and melting of the snowpack replaces water lost by sublimation of surface ice and evaporation in mid-summer. The basin contains no higher plants, but planktonic algae are very abundant [40].

The Cotton Glacier stream (CG) is a supraglacial stream in the Transantarctic Mountains (77°07′ S, 161°40′ E), flowing eastward between Sperm Bluff and Queer Mountain in Victoria Land for ~16 km (Figure 1). While the Cotton Glacier stream has a limited catchment area in the Clare and St. Johns ranges, it receives large amounts of sedimentary deposits of unknown origin (glacial or aeolian) from the surrounding areas. Water temperatures between 4.0–5.2 °C in stream beds with sediments have been reported [41].

### 2.2. Bacterial Isolation and Phylogenetic Characterization

The bacterial isolates used in this study (termed PL 17, PL 19, CG 23.3, and CG 23.4) were isolated from PL and the CG on R2A agar media (Difco) at 4 °C. Single colonies from each site were selected according to morphological characteristics, including color, size, and colony shape. Colonies were sub-cultured to obtain purified isolates. Isolates were stored in 40% glycerol at −80 °C and archived as part of the Foreman Lab Isolate Collection.

Genomic DNA was extracted using the PowerSoil DNA Isolation Kit (Qiagen, Valencia, CA, USA) and following manufacturer’s recommendations. PCR amplification of the 16S rRNA gene was performed with primers 9F 5′-GAGTTTGATCCTGGCTCAG-3′ and 1492R 5′-GGTTACCTTGTTACGACTT-3′ [42]. The PCR products were sent to Functional Biosciences (Madison, WI, USA) for Sanger sequencing. Sequences were assembled using the BioEdit 7.2 software package (Informer Technologies, Los Angeles, CA, USA). The nucleotide sequences were compared with the National Center for Biotechnology Information (NCBI) nucleotide database, using the Nucleotide Basic Local Alignment Search Tool (BLAST) search tool [43]. Sequences were aligned using the SILVA SINA 1.2.11 software package [44]. A maximum likelihood tree using the general time reversible model with 100 bootstraps was computed with the Molecular Evolutionary Genetics Analysis (MEGA-X) software program [45]. Sequences were deposited in the NCBI database under the accession number MT594460-MT594463.

### 2.3. Bacterial Growth

Bacterial isolates from freezer stocks were streaked on R2A agar plates and incubated at 4 °C. Single colonies of each organisms were transferred to 50 mL Falcon tubes containing 10 mL of modified R2A broth media (yeast extract = 0.5 g L^−1^, proteose peptone no. 3 = 0.5 g L^−1^, casamino acids = 0.5 g L^−1^, sodium pyruvate = 0.3 g L^−1^, dipotassium phosphate = 0.3 g L^−1^, magnesium sulfate = 0.05 g L^−1^). All liquid cultures were incubated at 4 °C while shaking at 100 rpm for 5–10 days at log phase. Cells were harvested by centrifugation at 4 °C and 4800 rpm for 15 min. Cell pellets were washed twice with 1X M9 Minimal Salts culture media (Difco™). Final cell pellets were resuspended in carbon-free 1X M9 Minimal Salts culture media.

#### Growth Media for Biosurfactant Production

To test for biosurfactant production, 10 mL of exponentially growing cell cultures were harvested and washed twice with 1× M9 Minimal Salts culture media. Final cell pellets were resuspended in 10 mL of 1× M9 Minimal Salts culture media supplemented with 0.2 µm filter sterilized canola oil (2% final concentration). Cell cultures were allowed to grow at 4 °C while shaking at 100 rpm for 10 days. Biosurfactant was extracted by centrifugation at 12,000× *g* for 5 min at 22 °C. The resulting supernatant was used for screening purposes.

### 2.4. Biosurfactant Screening Methods

#### 2.4.1. Oil Displacement Method

Oil displacement tests were carried out as described by Parthipan et al. [46]. Briefly, 40 mL of distilled water was added to a petri dish covered with 100 µL of crude oil. Then 10 µL of cell-free supernatant was pipetted onto the crude oil surface, and the diameter of the clear area was measured. Sterile growth media for biosurfactant production was used as a negative control. Solutions of sodium dodecyl sulfate (SDS; 0.05%–1.00% *w*/*v*), a known chemical surfactant, were used as a positive control.

#### 2.4.2. Emulsion Index Test (*E*_24_)

An emulsion index test was performed similar to the one originally developed by Cooper and Goldberg [47]. In it, 2 mL of supernatant was added to a test tube containing 2 mL of kerosene. The test tube was then vigorously vortexed for 2 min. Heights of emulsion, kerosene, and aqueous zones were measured after 24 h. The emulsions index (*E*_24_) was calculated by the following formula:

$$E_{24}=\frac{h_{emulsion}}{h_{total}}\times 100$$

where *h_emulsion_* and *h_total_* are the height of the emulsion layer and the total solution, respectively. Sterile growth media for biosurfactant production was used as a negative control, and 10% SDS was used as a positive control.

#### 2.4.3. Growth on Crude Oil

Media agar plates with 1× M9 Minimal Salts culture media were made and coated evenly with 100 µL of crude oil. Then, 1.5 mL of the cell culture growing in the R2A broth media was harvested by centrifugation and washed twice, as previously described. Cell pellets were resuspended in 100 µL of 1× M9 Minimal Salts culture media. The organisms were streaked on 1× M9 Minimal Salts culture media plates coated with 100 µL of crude oil as a sole carbon source and incubated at 4 °C. Plates were visually inspected for growth over a two-month period.

### 2.5. Biosurfactant Classification

#### 2.5.1. Large Scale Biosurfactant Production

To increase the amount of biosurfactants produced, two enrichment flasks containing 140 mL of growth media for biosurfactant production were prepared for each bacterial isolate. Cells were grown as described above. Cell cultures were pelleted by centrifugation at 4800 rpm for 15 min, and the supernatant was collected in pre-combusted (450 °C for 5 h) glass beakers. The beakers were dried in a drying oven at 80 °C for 72 h. The dried biosurfactant was scraped from the beaker into 50 mL falcon tubes. Subsequently, 10 mg of the dried biosurfactant were dissolved in either 1 mL of full-strength acetone, acetic acid, or ethanol at 37 °C for 24 h. To ensure the presence of dissolved biosurfactant in each solution, an oil displacement test, as described above, was performed, along with each solvent as a negative control. Biosurfactants were classified using matrix-assisted laser desorption/ionization (MALDI) mass spectrometry and Raman spectroscopy.

#### 2.5.2. MALDI Mass Spectrometry and Thin-Layer Chromatography

MALDI mass spectrometry was performed using a Bruker Autoflex MALDI TOF/TOF (Bruker Daltonik, Bremen, Germany), with ion source 1 voltage = −20.01 kV, ion source 2 voltage = −17.81 kV, reflector 1 = −21.25 kV, reflector 2 = −10.78 kV, and lens voltage = −8.70 kV. The MALDI TOF/TOF was equipped with a Smartbeam laser (Nd/YAG = 355 nm) capable of operating at a repetition rate of 1000 Hz. The MALDI laser used was capable of optimized delayed extraction time, and the laser beam size was set to small. For each sample preparation, the laser energy was optimized for an optimum signal-to-noise in the samples.

Thin-layer chromatography (TLC) was used to separate and confirm the presence of polar and non-polar (neutral) glycolipids following the method described by Nozomu and Makoto [48]. Briefly, 10 mg of dried, crude biosurfactants were dissolved in 1 mL CHCl_3_/MeOH (1:2, *v*/*v*). Samples were applied to TLC silica gel plates and developed in a neutral solvent system of CHCl_3_/MeOH/DIW (65/25/4, *v*/*v*/*v*, non-polar) and CHCl_3_/MeOH/DIW (90/9/1, *v*/*v*/*v*, strongly non-polar), respectively. Glycolipids were stained reversibly with iodine.

#### 2.5.3. Raman Spectroscopy

Raman spectroscopy was performed on a LabRAM HR Evolution Confocal Raman Spectrometer (HORIBA) equipped with the LabSpec6 software package (HORIBA, Japan). Dried surfactant samples (10 mg) were dissolved in 100 µL of deionized water. Then 6 µL of each dissolved sample was pipetted onto a clean CaF_2_ Raman-grade slide and placed in the drying oven at 40 °C for 1.5 min to evaporate off the deionized water. The samples were analyzed using a 100× objective with an NA of 0.9, and a 532 nm laser with 45 mW of power. The laser was operated at between 1–10% for three accumulations of 10 s for each spectrum. Measurements were recorded using a 600 nm grating. Spectra were fitted with a fourth-degree polynomial line for baseline subtraction. The LabSpec6 fourth-degree data smoothing function was used to reduce the noise-to-signal ratio of the measurements.

### 2.6. Respirometry

Bacterial isolates were further investigated for their hydrocarbon biodegradation potential at cold temperatures (i.e., 4 °C). A Columbus Instruments Micro-Oxymax Respirometer was used to continuously measure CO_2_ concentrations and CO_2_ production rates during extended incubations. Data were evaluated by Micro Oxymax software. Briefly, bacterial isolates were grown in R2A broth media, washed, and resuspended in 1X M9 Minimal Salts culture media with no carbon, as described above. The hydrocarbon amendments included diesel, motor oil (SUPERTECH, SAE5W-30), and crude oil, at a final concentration of 4.5% (*v*/*v*). Hydrocarbon amendments were added to combusted amber bottles in quadruplicate, containing 30 mL of 1X M9 Minimal Salts culture media and cells. One incubation bottle without cells served as an abiotic control for each amendment. All samples were incubated at 4 °C for 30 days, with data collected every 12 h. CO_2_ production rates were normalized to cell concentrations based on viable plate counts of colony-forming units (CFUs) on R2A agar and control corrected. The abiotic blanks were also investigated for any bacterial contamination at the end of each experiment via plate counts of CFUs on R2A agar. Unpaired *t*-tests and one-way ANOVA were performed using the statistical software package R [49], using a significance level of 0.05.

## 3. Results

### 3.1. Phylogeny of Biosurfactants Producing Bacterial Isolates

The phylogenetic characterization of the four bacterial isolates is summarized in Table 1. The closest identified relatives for CG 23.3 and CG 23.4 are reported as being psychrotrophic strains most closely related to *Janthinobacterium svalbardensis*. Although *Janthinobacteria* are commonly known to produce the purple pigment violacein, both isolates from this study did not produce purple colonies when grown on R2A agar plates. It is, however, not uncommon for species within this genus to lack the gene set required for the synthesis of the violacein pigment [50,51,52]. PL 19 was most closely related to *Psychrobacter arcticus* and was first isolated from Siberian permafrost [53]. PL 17 was the only isolate related to mesophilic phylotypes. Its closest relatives were all from the genus *Serratia* (>99%). Some slightly more distantly related relatives (>97%) were also reported as being psychrotrohic isolates [54].

### 3.2. Screening for Biosurfactants

All four bacterial isolates possessed emulsifying activity (Table 2). Emulsification indexes, *E*_24_, were recorded between ~36% (*Serratia* sp. PL 17) and 67% (*Janthinobacterium* sp. CG 23.3), which bracketed the *E*_24_ index for a 10% SDS solution (~56%). The addition of the surfactant supernatant to a crude oil layer for the oil displacement test formed average clear zones of ~3.5 ± 2.2 cm (*Janthinobacterium* sp. CG 23.4) to ~6.4 ± 0.4 cm (*Serratia* sp. PL 17) in size (Table 2), which were comparable to the performance of a 0.1% (2.7 ± 0.1 cm) to 1.0% SDS (6.7 ± 0.4 cm) solution, respectively. The amount of dried supernatant produced ranged between 8.5–17.3 g L^−1^. When grown on crude oil as a sole carbon source, excellent growth was observed for *Serratia* sp. PL 17. Colonies were also evident for *Janthinobacterium* sp. CG 23.4, and growth was visible for *Janthinobacterium* sp. CG 23.3, albeit much less pronounced. *Psychrobacter* sp. PL 19 did not form colonies on crude oil (Table 2).

### 3.3. Characteristics of Biosurfactants

**Figure 2:**
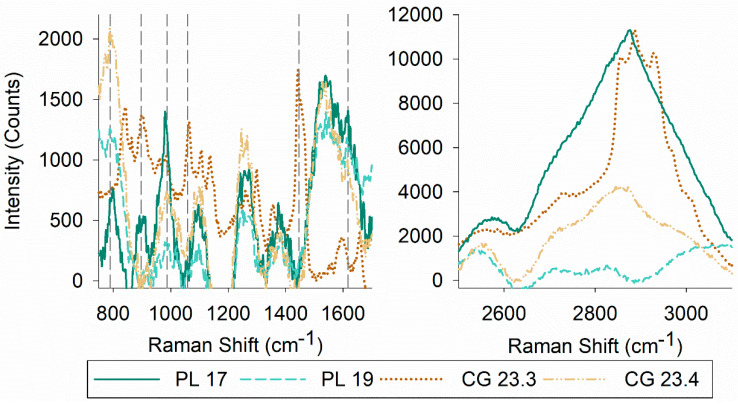
Raman spectra of biosurfactants: (**left panel**) wavenumbers from 750–1700 cm^−1^ and (**right panel**) from 2500–3100 cm^−1^. Dashed lines indicate peaks at specific wavenumbers associated with biosurfactants.

MALDI mass spectrometry data were analyzed to identify biosurfactants produced by the four bacterial isolates. Putative identification showed the presence of different sophorolipids (*m*/*z*: 621 (C_18:1_), *m*/*z*: 647 (C_18_ + Na), *m*/*z*: 663 (C_18:1_ 1Ac)), and di-rhamnolipids (*m*/*z*: 649 (Rha2 C_10_–C10), *m*/*z*: 689 (Rha2 C_12_–C_12_)) (Table 2). The presence of glycolipids was confirmed by TLC (Supplemental Figure S1). TLC also captured compositional differences of both polar and non-polar species in the biosurfactant extracts. Of the four biosurfactants, glycolipids produced by *Serratia* sp. PL 17 and *Psychrobacter* sp. PL 19 were the most polar and non-polar, respectively. A commonality was found between the prominent TLC band for both *Janthinobacterium* spp. CG 23.3 and CG 23.4 (Supplemental Figure S1). As biosurfactants are a mixture of compounds rather than a pure substance, Raman spectroscopy was used to gain additional information on the structural composition of the biosurfactants (Figure 2).

The most characteristic features of the Raman spectra of lipids are related to stretching vibrations. Raman spectra also differ based on functional groups of lipids and the structure of a given molecule (e.g., saturation, phase, solubility) [57]. Raman spectra yielded peaks at 789 cm^−1^ (*Serratia* sp. PL 17, *Psychrobacter* sp. PL 19, and *Janthinobacterium* sp. CG 23.4), 897 cm^−1^ (*Serratia* sp. PL 17 and *Janthinobacterium* sp. CG 23.3), 987 cm^−1^ (*Serratia* sp. PL 17, *Psychrobacter* sp. PL 19, *Janthinobacterium* sp. CG 23.3, and *Janthinobacterium* sp. CG 23.4), 1058 cm^−1^ (CG 23.3), 1446 cm^−1^ (*Janthinobacterium* sp. CG 23.3), and 1617 cm^−1^ (*Serratia* sp. PL 17, *Psychrobacter* sp. PL 19, and *Janthinobacterium* sp. CG 23.4), which is indicative of (CS) vibration of aliphatic chains, (OO) stretching, (CC) alicyclic/aliphatic chain vibrations, (CC) stretching vibrations, alkyl CH_2_ bends, and amide I peaks, respectively. In the higher wavenumber range (~2800–3100cm^−1^), differences in the Raman spectra for each biosurfactant were evident, which can be assigned to the CH stretching of acyl or alkyl chains (Figure 2).

### 3.4. Respiration Activity

**Figure 3:**
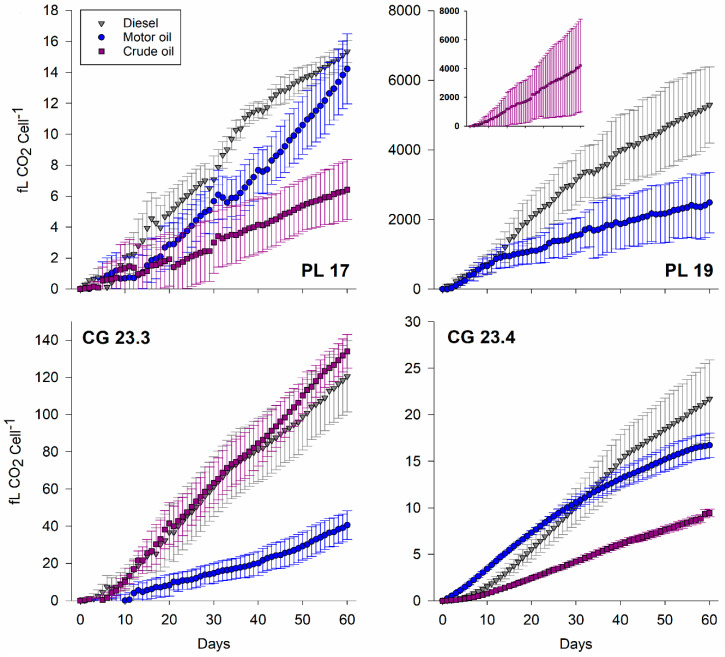
Cumulative carbon dioxide production during biodegradation of diesel, motor oil, and crude oil. Each data point represents the average and standard deviation of four replicates (blank corrected).

The four bacterial isolates were able to grow on diesel, motor oil, and crude oil as their sole carbon and energy source. No growth was observed in the controls when grown on R2A agar plates after 30 days of incubation. Respiratory activity is shown by the cumulative CO_2_ production continuously monitored over 30 days (Figure 3). The biodegradation of the three substrates generally resulted in a linear increase of CO_2_ production (*r*^2^ ≥ 0.971). Rates varied markedly over two orders of magnitude between isolates, even within the same species. The highest respiration rates were found for *Psychrobacter* sp. PL 19 for all three hydrocarbon substrates (Table 3); these rates were significantly higher compared to the other bacterial isolates (two-sided *t*-test; *p*-value ≤ 0.038). It should be noted that when grown on R2A broth at 4 °C, the generation time of the bacterial isolates ranged between 5.0–6.5 h (*Serratia* sp. PL 17: 6 h 02 min, *Psychrobacter* sp. PL 19: 6 h 02 min, *Janthinobacterium* sp. CG 23.3: 5 h 04 min, and *Janthinobacterium* sp. CG 23.4: 6 h 21 min), which further underlies the petroleum hydrocarbon degradation capabilities of *Psychrobacter* sp. PL 19. However, although CO_2_ production rates for *Psychrobacter* sp. PL 19 varied between 344.0 ± 81.5 fg CO_2_ d^−1^ cell^−1^ for diesel, 144.8 ± 59.7 fg CO_2_ d^−1^ cell^−1^ for motor oil, and 271.6 ± 199.1 fg CO_2_ d^−1^ cell^−1^ for crude oil, they were not statistically different (one-way ANOVA, F_(2, 9)_ = 2.445, *p*-value = 0.142).

## 4. Discussion

The four biosurfactant producing bacterial strains were isolated from ephemeral Antarctic environments. It is well-established that many microorganisms produce tensio-active molecules, including biosurfactants, in order to increase the bioavailability of hydrophobic, water-insoluble substances, or the hydrophobicity of the cell surface [11,13,17]. However, none of the Antarctic environments are known to have experienced large accidental releases of hydrocarbons, although potential small-scale contamination linked to human activities cannot be ruled out. Given the lack of evidence for petroleum hydrocarbon contaminants, the intrinsic roles of biosurfactants in these environments may be antimicrobial activity as a competitive strategy, oxidative stress protection, and switching from sessile to planktonic lifestyles and vice versa in response to nutrient availability [11,17,58].

Putative identification suggests that all four isolates produced sophorolipids and di-rhamnolipids when grown on canola oil as a sole carbon source (Table 2). Noteworthily, diglycerides with two oleic acid residues, which are likely abundant in canola oil and present in the dried, crude biosurfactant, exhibit the same *m*/*z* ratios as certain sophorolipids (*m*/*z*: 621) and di-rhamnolipids (*m*/*z*: 647, 649). Hence, caution is advised when generalizing the putative classification of sophorolipids and rhamnolipids in this study. As sophorolipid production has only been observed in yeast to date [11], these biosurfactants should be subject to more detailed characterization. Rhamnolipids are glycolipids, which contain one or two rhamnose molecules (i.e., mono-rhamnolipids and di-rhamnolipids, respectively) as the sugar moiety linked to β-hydroxylated fatty acid chains [59]. Putative identification of rhamnolipids showed a pronounced peak for di-rhamnolipid C_12_–C_12_ for all four bacterial isolates, while other rhamnolipid peaks could have been masked by the noise of the measurement. An additional peak indicative of di-rhamnolipids C_10_–C_10_ was apparent for the bacterial isolate *Serratia* sp. PL 17. Naturally-produced rhamnolipid biosurfactants are found as mixtures of different congeners, and as such, the chemical composition of the dominant rhamnolipids and the concentration of the congeners largely determines the efficacy of the biosurfactant. Generally, a higher di-rhamnolipid content enhances biosurfactant activity [60,61]. The predominance of putatively identified di-rhamnolipid C_10_–C_10_ and di-rhamnolipid C_12_–C_12_ implies that the physicochemical properties of the biosurfactants produced by the four Antarctic isolates could be up to ~8 and 45 times more effective when compared to mono-rhamnolipids with C_10_ fatty acids or rhamnolipid mixtures with high proportions of congeners containing unsaturated fatty acids, respectively [59]. Considering that ~60 rhamnolipid congeners and homologues with different chemical structures and surface properties have been identified [27], it is very likely that specific groups of biosurfactants could provide an advantage in different ecological niches. Whether these prominent di-rhamnolipids are particularly suited to functioning at low temperatures remains, however, unanswered, due to the dearth of information on biosurfactant producing microbes from cold environments.

All biosurfactants produced at 4 °C showed emulsification. The bacterial isolates *Psychrobacter* sp. PL 19 and *Janthinobacterium* sp. CG 23.3 could significantly emulsify kerosene (*E*_24_ > 50%), with a maximum index of 66.7% for *Janthinobacterium* sp. CG 23.3 (Table 2). The emulsifying ability of both isolates was bracketed by *E*_24_ indexes for light mineral oil, tetradecane, and sunflower oil reported from excellent biosurfactant producers (*Rhodococcus* spp., 50–67%) isolated from marine Arctic and Antarctic environments [62,63]. *Pseudomonas aeruginosa* and *Bacillus subtilis*, two bacterial species that have traditionally been considered for biosurfactant production, have *E*_24_ values of ~70% and ~10% for kerosene, respectively [64]. Dried biosurfactant supernatants of *Psychrobacter* sp. PL 19 (13.3 g L^−1^) and *Janthinobacterium* sp. CG 23.3 (17.3 g L^−1^) also indicate biosurfactant production similar to those reported for the yeast *Moesziomyces antarcticus* (16.3 g L^−1^). *M. antarcticus* (previously classified as *Pseudozyma antarctica*), which was isolated from Lake Vanda, Antarctica [65], is one of the best-known producers of glycolipid biosurfactants [66]. Overall, biosurfactant production was one to two orders of magnitude higher compared to other microorganisms from low-temperature environments [28]. However, without standardized methodologies for the field, such direct comparisons are complex, as different conditions (i.e., optimal vs. suboptimal hydrocarbon substrates, nutrient concentrations, physico-chemical parameters) used in these types of assays largely affect the strength of emulsification activity and biosurfactant production rates [67]. Further optimization of the biosurfactant production capabilities of the organisms will aid the biotechnological exploration.

The bacterial isolates *Psychrobacter* sp. PL 19, *Janthinobacterium* sp. CG 23.3, and *Janthinobacterium* sp. CG 23.4 were closely related to *Psychrobacter* sp. and *Janthinobacterium* spp., respectively (Table 1). While both genera are commonly found in hydrocarbon-contaminated environments, hydrocarbon substrates have only been shown to enrich and select for different *Psychrobacter* phylotypes [68,69,70]. Janthinobacteria, on the other hand, are believed to tolerate petroleum hydrocarbon toxicity rather than using oil components as carbon sources [71], although, indirect measurements (i.e., community structure analysis of contaminated soils based on 16S rRNA genes) hint at their hydrocarbon degrading capabilities [72,73]. Likewise, the petroleum hydrocarbon degradation activity of *S. quinivorans*, which was most closely related to *Serratia* sp. PL 17, based on 16S rRNA gene similarity, has not previously been demonstrated. Thus far, primarily different *S. marcescens* strains have been investigated [74,75].

All four isolates grew on diesel, motor oil, and crude oil (Figure 3). With different approaches being described across the literature to quantify petroleum hydrocarbon degradation, direct comparisons are not feasible. The potential of these bacterial isolates to grow on petroleum hydrocarbons as their sole carbon source clearly represents fertile territory for further study and biotechnological exploration.

## 5. Conclusions

Biosurfactants have emerged as viable solutions in many industrial applications, with the potential to outperform traditional synthetic surfactants due to their unique, environmentally friendly properties. Bioprospecting cold-adapted microbes for new biosurfactants has been limited, with established reports being rather incomplete in terms of identified strains and biosurfactants characterized [39,67]. Other biotechnological hurdles to overcome are related to inefficient biosurfactant production methods that are competitive at a marketable scale. In their review “Rhamnolipids: diversity of structures, microbial origins and roles”, Abdel-Mawgoud et al. [27] emphasize that the ability to produce rhamnolipids appears to be restricted to a limited number of bacterial species, and that many reports are simply anecdotal. More recently, Irorere et al. [67] state that numerous studies have insufficient evidence to support claims of rhamnolipid-producing strains. The present study explored the biosurfactant production of Antarctic bacterial isolates, revealing two isolates closely related to *Janthinobacterium*, a genus previously not known for biosurfactant production. Moreover, putative biosurfactant identification for all isolates produced pronounced peaks indicative of di-rhamnolipids. The discovery of new biosurfactant producing species merits further investigations, including molecular and bioinformatic tools, in order to unambiguously classify these organisms and their biosurfactant products. Current efforts await the in silico functional profiling of the draft genomes of the four bacterial isolates and biosurfactant production optimization (i.e., pH, temperature, and carbon and nutrient sources). Further research on petroleum hydrocarbon degradation should include gravimetric methods and calibrated analytical measurements (e.g., high-performance liquid chromatography or ultra-performance liquid chromatography) for optimal quantification [67].

## Figures and Tables

**Table 1:** Antarctic bacterial isolates and their closest relatives based on 16S rRNA gene sequence identity.

Isolate	Location	Closest Relative	% ID	Accession #
PL 17	Pony Lake	*Serratia quinivorans* 4364 (NR_037112.1)	99	MT594460
PL 19	Pony Lake	*Psychrobacter arcticus* strain 273-4 (NR_075054.1)	98	MT594461
CG 23.3	Cotton Glacier	*Janthinobacterium svalbardensis* JA-1 (NR_132608.1)	98	MT594462
CG 23.4	Cotton Glacier	*Janthinobacterium svalbardensis* JA-1 (NR_132608.1)	99	MT594463

**Table 2:** Biosurfactant activity and putative identification.

Isolate	*E*_24_ (%)	Oil Displacement (cm)	Dried Supernatant (g/L)	Growth on Crude Oil	MALDI Peak Matches (*m*/*z* Ratio)
PL 17	36.4	6.4 ± 0.4	8.5	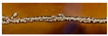	Sophorolipid (621, 663) ^i^ Di-rhamnolipid (649, 689) ^i,ii^
PL 19	58.3	3.5 ± 0.1	9.9	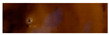	Sophorolipid (663) Di-rhamnolipid (689)
CG 23.3	66.7	4.6 ± 1.1	17.3	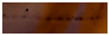	Sophorolipid (647) ^iii^ Di-rhamnolipid (689)
CG 23.4	46.2	3.5 ± 2.2	13.3	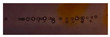	Sophorolipid (663) Di-rhamnolipid (689)

^i^ Marchant and Banat [9]; ^ii^ Nitschke et al. [55]; ^iii^ Lindum et al. [56].

**Table 3:** Degradation rates of hydrocarbon-containing substrates.

Isolate	Diesel (fg CO_2_ d^−1^ cell^−1^)	Motor Oil (fg CO_2_ d^−1^ cell^−1^)	Crude Oil (fg CO_2_ d^−1^ cell^−1^)
PL 17	1.0 ± 0.1	0.9 ± 0.2	0.0 ± 0.1
PL 19	344.0 ± 81.5	144.8 ± 59.7	271.6 ± 199.1
CG 23.3	7.8 ± 1.2	2.5 ± 0.5	8.7 ± 0.5
CG 23.4	1.5 ± 0.3	1.1 ± 0.1	0.6 ± 0.04

## Supplementary Materials

The following are available online at https://www.mdpi.com/2076-2607/8/8/1183/s1, Supplemental Figure S1: Thin-layer chromatograph results.
