# Supplementary material for: Low-Temperature Biosurfactants from Polar Microbes
Source: Microorganisms. 2020 Aug 3;8(8):1183. doi: 10.3390/microorganisms8081183 (PMC7466143; doi:10.3390/microorganisms8081183)
Supplement: Supplementary file 1 [file microorganisms-08-01183-s001.pdf]

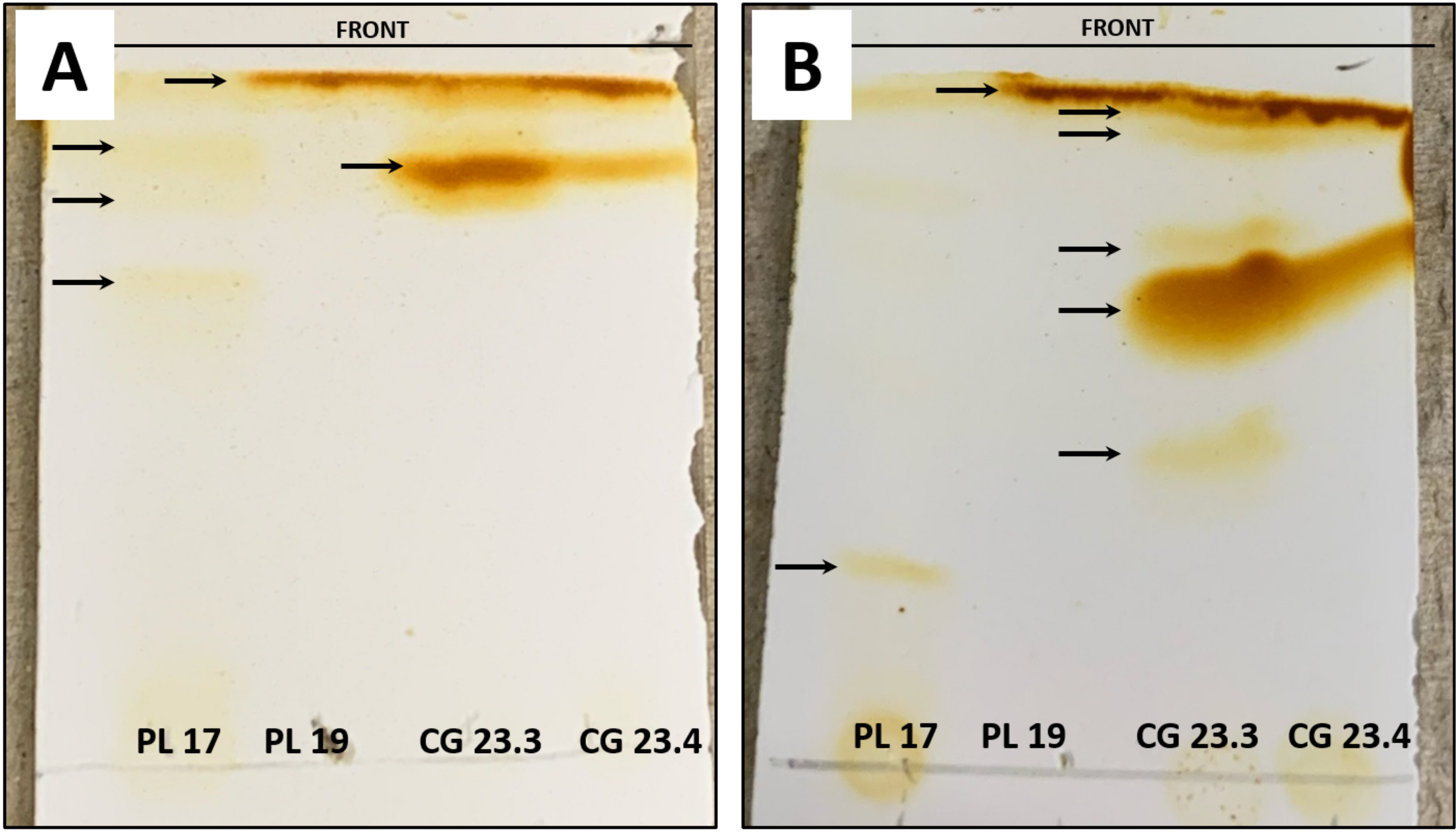

Supplemental Figure S1: TLC chromatograms of biosurfactants produced by *Serratia* sp. PL 17, *Psychrobacter* sp. PL 19, *Janthinobacterium* sp. CG 23.3, *Janthinobacterium* sp. CG 23.4. Plates were developed by ascending chromatography in a neutral solvent system of (A) $CHCl_3$/MeOH/DIW (65/25/4, v/v/v) and (B) $CHCl_3$/MeOH/DIW (90/9/1, v/v/v). Glycolipids were visualized using iodine. Arrows indicate separated glycolipids.
